# Supplementary material for: Manipulating single excess electrons in monolayer transition metal dihalide
Source: Nat Commun. 2023 Jun 21;14:3691. doi: 10.1038/s41467-023-39360-1 (PMC10284909; doi:10.1038/s41467-023-39360-1)
Supplement: Supplementary file 1 — Supplementary Information [file 41467_2023_39360_MOESM1_ESM.pdf]

Supporting information for

Manipulating single excess electrons in monolayer transition metal dihalide

Min Cai[1], Mao-Peng Miao[1], Yunfan Liang[2], Zeyu Jiang[2], Zhen-Yu Liu[1], Wen-Hao Zhang[1,3], Xin Liao[1], Lan-Fang Zhu[1], Damien West[2], Shengbai Zhang[2], and Ying-Shuang Fu[1,3,4*]

1. School of Physics and Wuhan National High Magnetic Field Center, Huazhong University of Science and Technology, Wuhan 430074, China
2. Department of Physics, Applied Physics and Astronomy, Rensselaer Polytechnic Institute, Troy, NY, 12180, USA
3. Institute for Quantum Science and Engineering, Huazhong University of Science and Technology, Wuhan 430074, China
4. Wuhan Institute of Quantum Technology, Wuhan 430206, China

*yfu@hust.edu.cn

SUPPLEMENTARY NOTES

1. STM contrast for different types of polarons

In principle, polarons should be visible if their presence perturbs the local density of states at the polaron sites. STM images of the polarons that appear as depressions or protrusions, depending on their electronic states. Since all kinds of polarons are electron type, they all induce upward local band bending, which allow them to be visualized as depressions at energies associated with the band bending, because their presence deleted the local density of states. At negative bias, type-II polarons are visualized as protrusions at energies of their polaronic states, but type-I (down) and type-I (up) polarons become invisible because their polaronic states are not discernible.

To depict the dependence of STM contrast of different polarons on their electronic states, we show a data set in Supplementary Fig. 8 as a typical example. Supplementary Fig. 8(a) is an STM

image of three polarons with different types at 1.2 V. The type-I (down) and type-I (up) polaron appear as a shallow and deep depression, respectively. However, the type-II polaron appears as a protrusion. Line spectra surpassing all the three kinds of polarons indicate that the type-I (up) polaron has more depleted electron density at 1.2 V than that of the type-I (down) polaron, and the type-II polaron has enhanced electron density compared to the polaron-free region [Supplementary Figs. 8(b, c)].

2. Localized polaronic states

Our calculations reveal a very localized (narrow) charge distribution of polaronic states along the out-of-plane direction (z-direction), due to the d-orbital nature of these states. As the STM tip is ~5 Å higher than the top Cl layer in experiments, much larger than the extent of 3d-orbitals, the sample-to-tip tunneling is dominated by the wavefunction “tail” of polaronic states, which decays exponentially into the surface region. Supplementary Fig. 13 shows the charge density of Co- and Cl-centered polarons along a line in the out-of-plane direction passing through the polaron center. Here it can be seen that while the charge densities of both states decay exponentially in the vacuum region, the charge density associated with the Cl-center polaron (and hence the tunneling current) is orders of magnitude larger that of the Co-centered polaron. As such, in-gap states associated with type-I (down) and type-I (up) polarons (Co-centered) are inherently more difficult to see from STS than the type-II polaron (Cl-centered).

3. Model of tip electric field

We modelled the electric field between the tip and the substrate, which were considered as a point charge and a semi-infinite metal, respectively, for simplicity. The metallic substrate creates

an image charge with opposite polarity in the presence of the point charge [Fig. 5(a), inset]. The electric filed of the surface location beneath the tip reads $E = \frac{Q}{2\pi\varepsilon_0(\Delta z + z_0)^2}$, where the tip charge $Q = C(V + \Delta W)$ with the capacitance between tip and sample C, the applied voltage V, and the work function difference between tip and sample ΔW. Z_0 and Δz represent the initial tip height and decreasing tip height, respectively. In the situation of constant critical electric field, the above relation gives $\mathrm{V} = \mathrm{k}(\Delta z + z_0)^2 - \Delta W$, where $\mathrm{k} = \frac{4\pi\varepsilon_0 E}{C}$ is a proportional constant.

SUPPLEMENTARY TABLE

Polaron conversion conditions

Type-I (down)→ Type-I (up)	Type-I (up)→Type-I (down)	Type-I (up)→Type-II	Type-II→Type-I (up)
3 V/5 nA	- 1.7 V/4 nA	- 1.7 V/4 nA	2.8 V/ 0.1 nA

SUPPLEMENTARY FIGURES

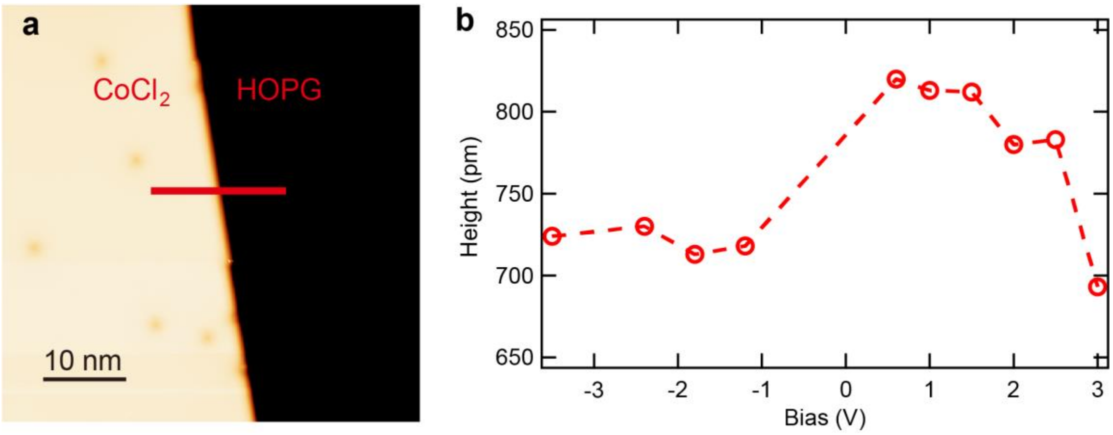

Supplementary Fig. 1 Bias dependent apparent height of monolayer $CoCl_2$ on HOPG. (**a**) STM image (V_s = 2 V, I_t = 10 pA) of monolayer $CoCl_2$ on HOPG substrate. (**b**) Measured apparent height for monolayer $CoCl_2$ against different sample bias.

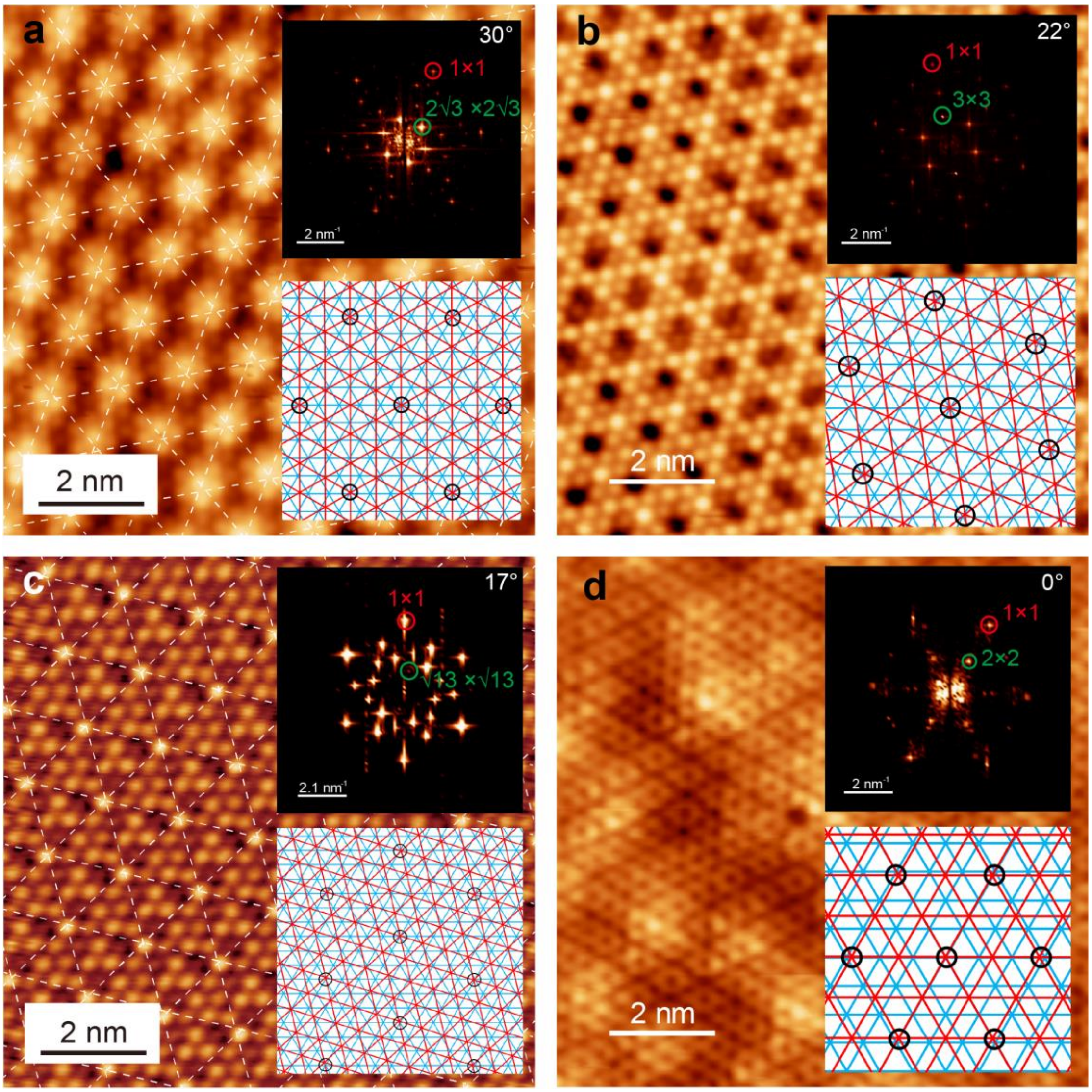

Supplementary Fig. 2 Moiré patterns between $CoCl_2$ film and HOPG. (**a-d**) STM images of $CoCl_2$ films showing different moiré patterns. The stacking angles between $CoCl_2$ and HOPG is marked. Fast Fourier transformations to the STM images and their lattice models are shown in the inset. The reciprocal spots for the top layer Cl lattice and the moire lattice are marked with red and green circles, respectively. In the lattice models, the blue (red) lines represent the HOPG ($CoCl_2$) lattice with a lattice constant of 0.246 nm (0.35 nm). Imaging conditions: (**a**) V_s = 0.5 V, I_t = 50 pA; (**b**) V_s = 0.3 V, I_t = 50 pA; (**c**) V_s = - 0.3 V, I_t = 20 pA; (**d**) V_s = - 0.6 V, I_t = 50 pA. The regular moiré patterns demonstrate no strain are exerted on the film from the substrate.

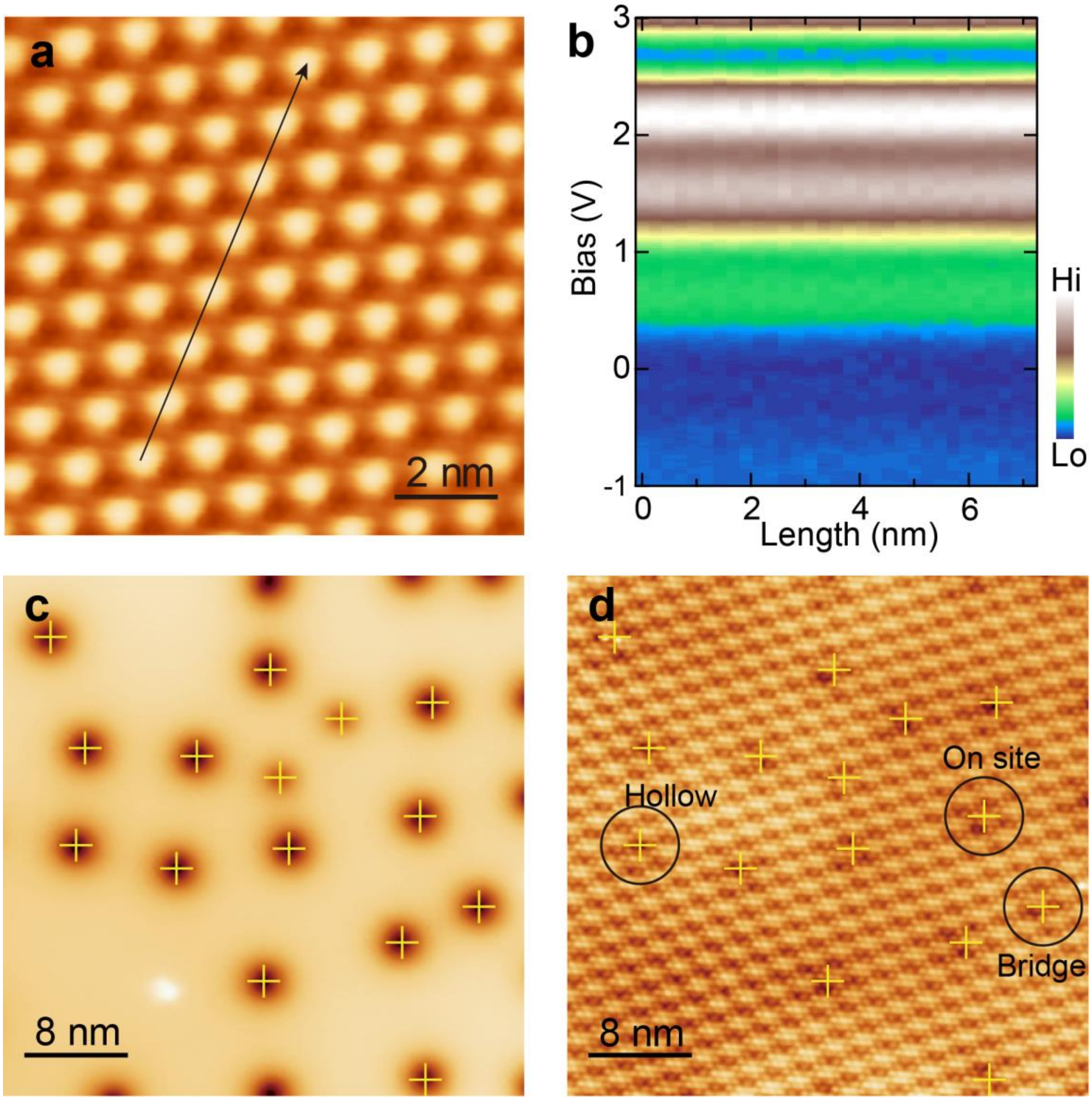

Supplementary Fig. 3 Relation between the polaron distribution and the moiré pattern of $CoCl_2$. (**a**) STM image ($V_s = 0.2$ V, $I_t = 10$ pA) showing a $\sqrt{13} \times \sqrt{13}$ moiré superlattice on polaron-free area. (**b**) 2D conductance plot taken along the black arrow in (**a**). Spectroscopic condition: $V_s = 3$ V, $I_t = 100$ pA, $V_{mod} = 40$ mV. (**c, d**) STM images at the same field of view showing the polaron locations (marked with yellow crosses) and moiré patterns, respectively, with different imaging conditions ($V_s = 1.2$ V, $I_t = 10$ pA for c; $V_s = 0.3$ V, $I_t = 50$ pA for d). There is no correlation between the polaron sites (yellow crosses) and the moiré pattern.

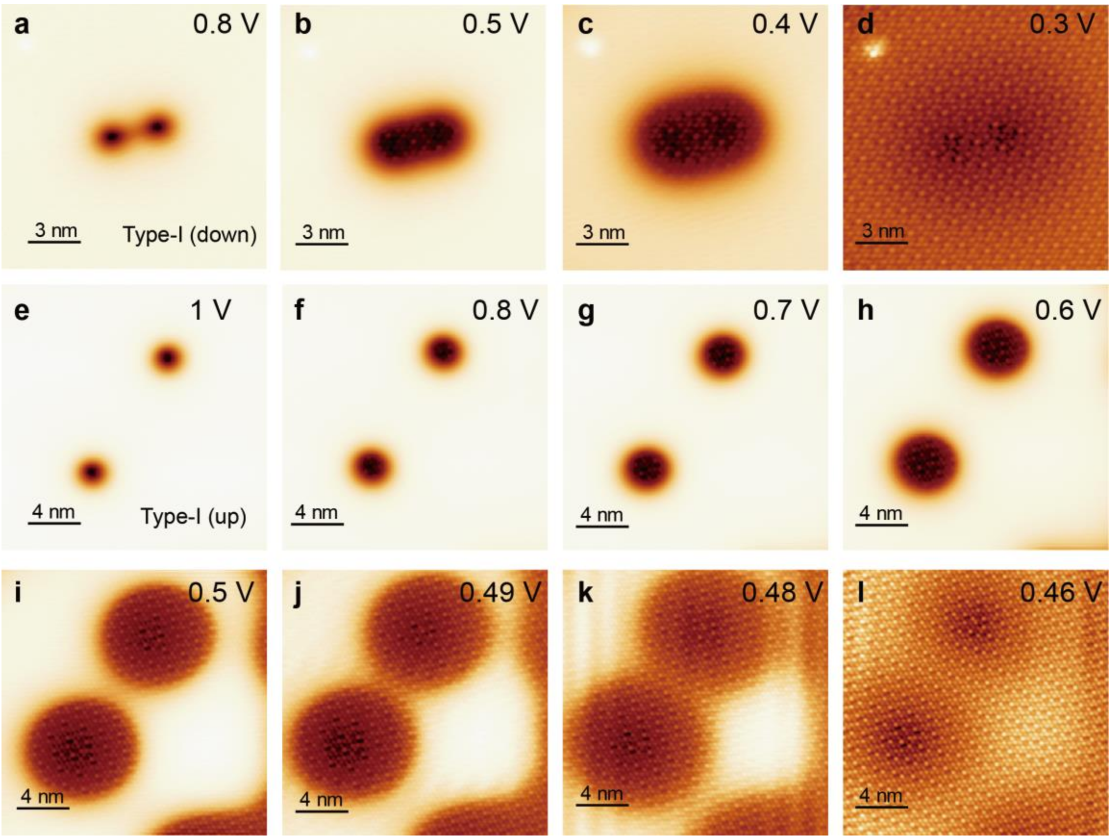

Supplementary Fig. 4 Bias dependent STM images of the type-I (down) and type-I (up) polarons. (**a-d**) STM images of type-I (down) polarons. (**e-l**) STM images of type-I (up) polarons. The corresponding sample biases are marked.

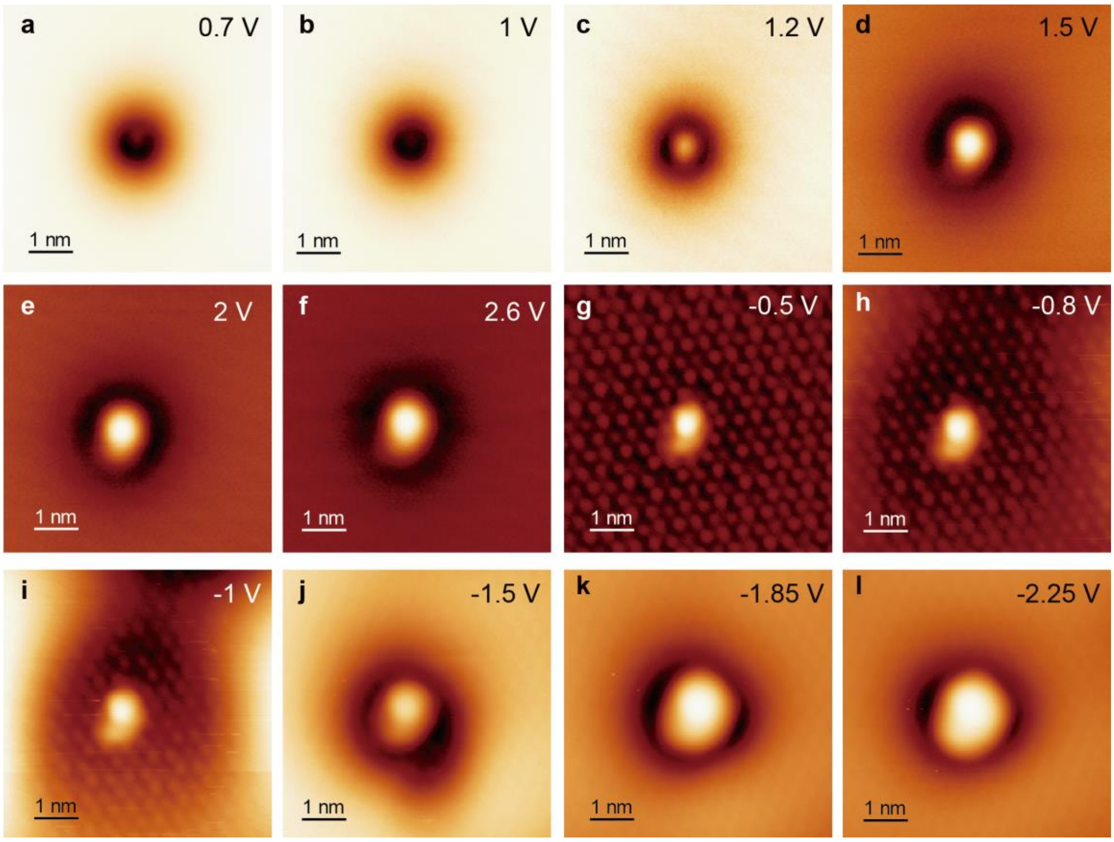

Supplementary Fig. 5 STM topography of type-II polaron at different voltages. The type-II polaron appears as a protrusion above 1.2 V and below -0.5 V. Note that the slight tail of the protrusion is caused by the imperfect shape of the tip apex.

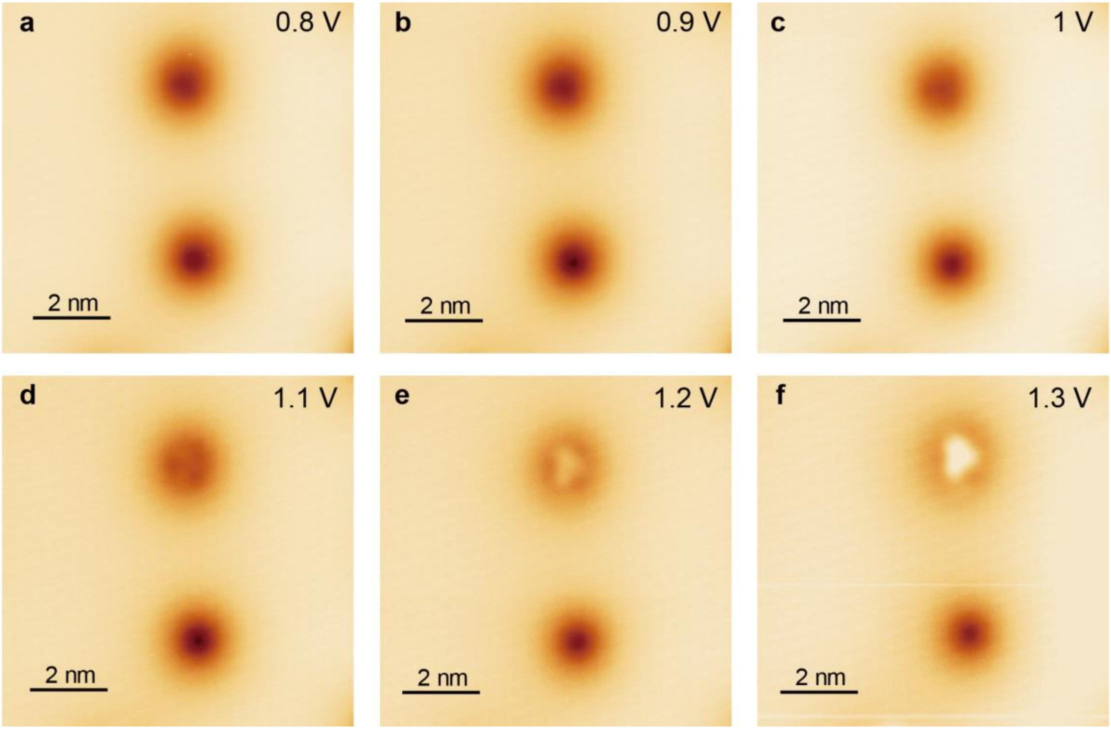

Supplementary Fig. 6 Bias dependent STM images of type-I (down) and type-II polarons. The scanning biases are marked. These two types of polarons look similar as depressions at the scanning bias lower than 1 V, while the type-II polarons appear as protrusions when the scanning bias exceeds 1 V.

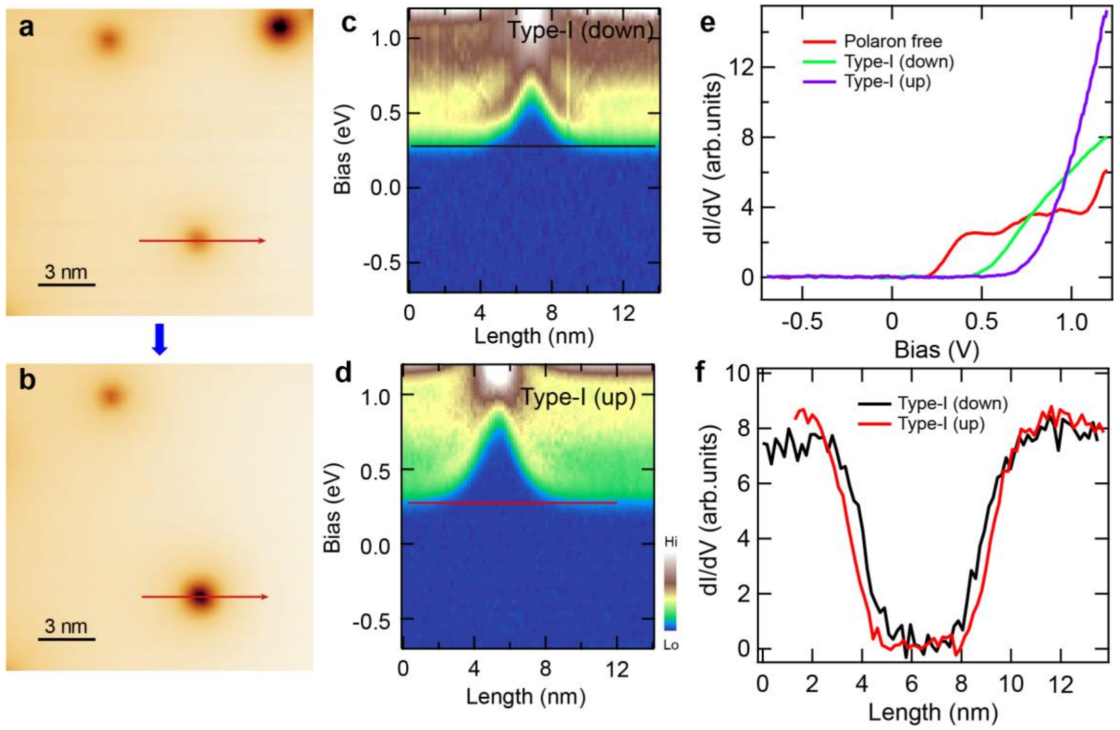

Supplementary Fig. 7 Comparison of the band bending between type-I (down) and type-I (up) polarons. (**a, b**) STM images (V_s = 1 V, I_t = 10 pA) taken before and after converting a type-I (down) polaron to a type-I (up) polaron. (**c**)[(**d**)] 2D conductance spectra measured across the type-I (down) and type-I (up) polaron along the red line in (**a**) [(**b**)] respectively. Spectroscopic condition: V_s = 1.2 V, I_t = 200 pA. (**e**) Point spectra taken at polaron-free area, type-I (down) polaron center and type-I (up) polaron center. The degree of upward band bending for the type-I (up) polaron is larger than that of the type-I (down) polaron. (**f**) dI/dV profile taken at energy of the conduction band edge indicated with red and black lines in (**c**) and (**d**), showing that type-I (down) and type-I (up) polaorns have similar radii.

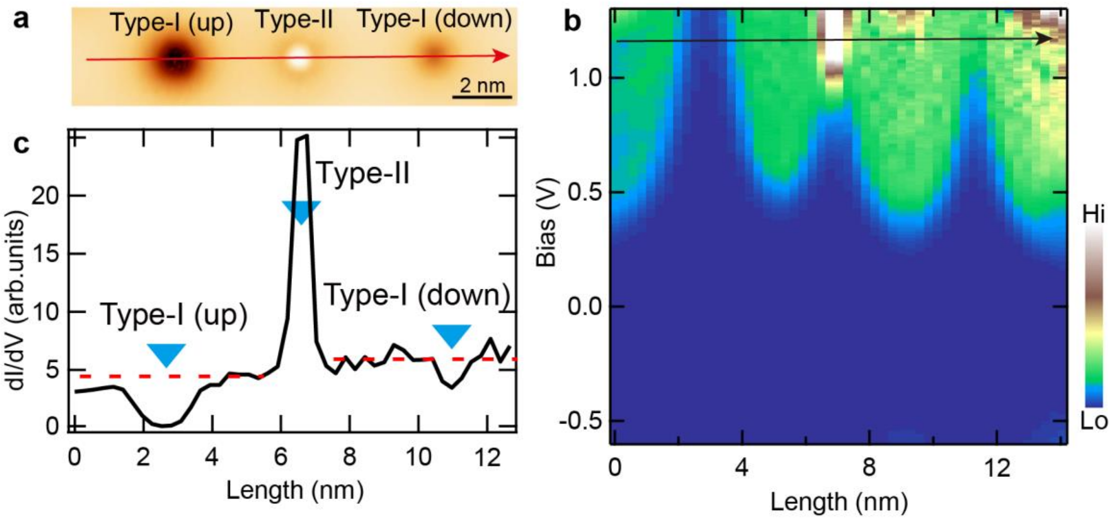

Supplementary Fig. 8 STM image of three kinds of polarons showing different contrast. (**a**) STM image (V_s = 1.2 V, I_t = 10 pA) of three kinds of polarons. (**b**) Constant height 2D conductance spectra taken through three kinds of polarons along the red arrow in (**a**) (V_s = 1.3 V, I_t = 150 pA, V_{mod} = 20 mV). (**c**) Spectroscopic profile taken along the black arrow in (**b**) (V_b = 1.2 V). The red dashed line indicates the conductance intensity at polaron-free area. The position of the three kinds of polarons are marked with cyan triangles.

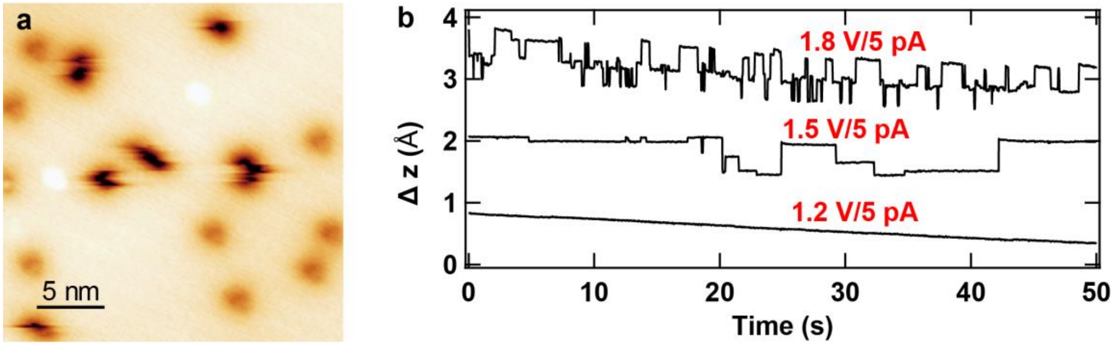

Supplementary Fig. 9 Stability of type-I (down) and type-I (up) polarons. (**a**) STM image (V_s =2.5 V, I_t =10 pA) taken at 2.5 V. Type-I (down) polarons are stationary while type-I (up) polarons are affected by the scanning, manifesting the different stability of these two types of polarons. (**b**) Time traces of the instability of tip height (Δz) recorded over a single type-I (up) polaron with different set point. The tip height switches at different values and the frequency becomes larger with increasing sample bias. Such tip instability of the type-I (down) polaron is similar but with higher sample bias and larger tunneling current (3 V/3 nA).

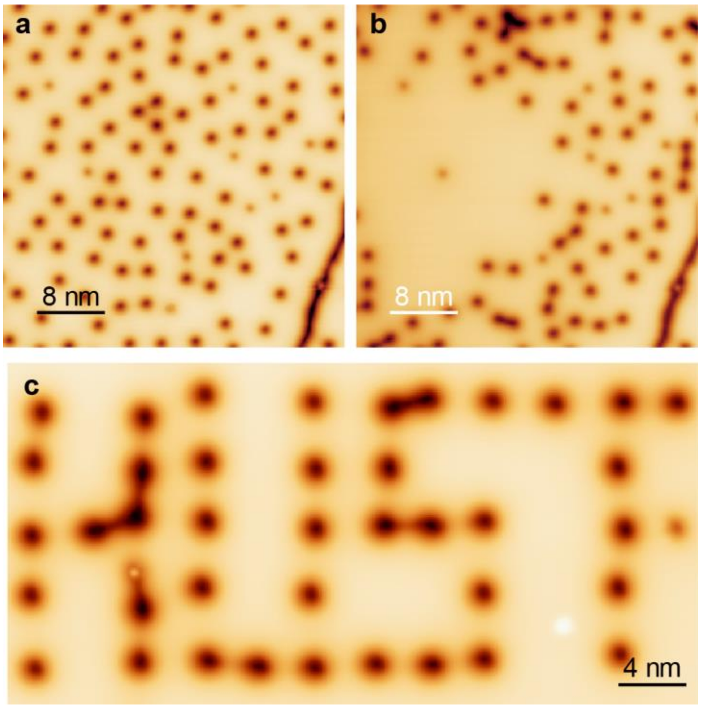

Supplementary Fig. 10 STM images (V_s =1.2 V, I_t =10 pA) showing a sequence of polaron manipulations. (**a,b**) STM images before (a) and after (**a**) manipulations to locally change the polaron density. The manipulating conditions are 3 V/3 nA for the type-I (down) polarons and 1.8 V/10 pA for the type-I (up) polarons, respectively. (**c**) STM image of manipulated polarons forming a designed pattern of "HUST", which is the abbreviation of Huazhong University of Science and Technology.

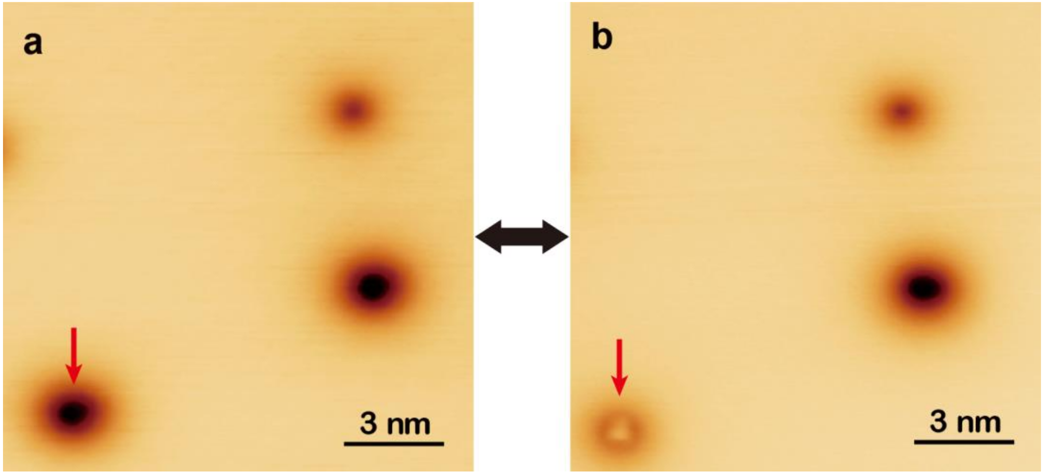

Supplementary Fig. 11 STM images (V_s =1.2 V, I_t =10 pA) showing interconversion between type-I (up) and type-II polarons. The red arrows mark the manipulated polarons.

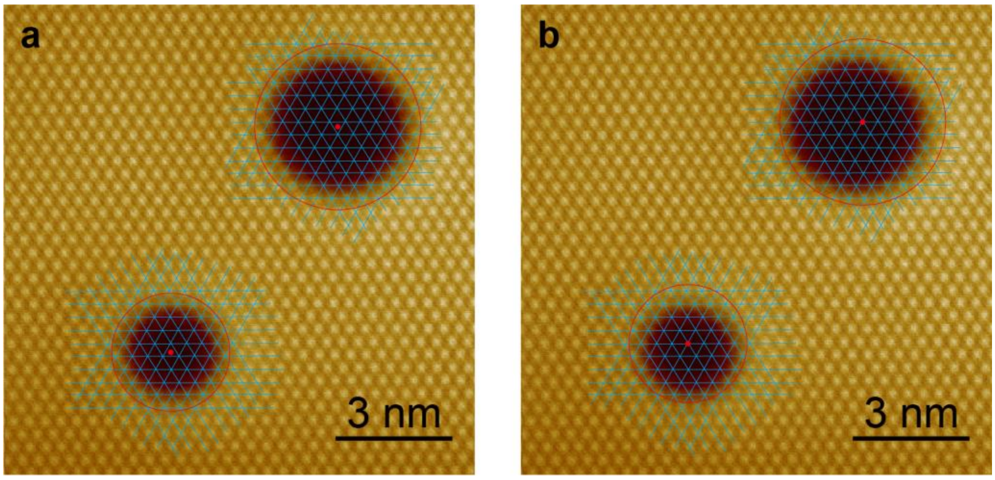

Supplementary Fig. 12 Atomic sites for the type-I (down) and type-I (up) polarons. (**a**) d*I*/d*V* map (V_s = 0.75 V, I_t = 10 pA) of a type-I (down) polaron (bottom) and a type-I (up) polaron (top). Atomic lattice of Cl layer superimposed on dI/dV map, showing centers of both kinds of polarons are determined as Co-site. The red circles mark the rings associated with band bending caused by the polarons. (**b**) The same as (**a**), but with deliberately positioning the ring centers on Cl-site. This obviously causes an ill-match to the polaron centers.

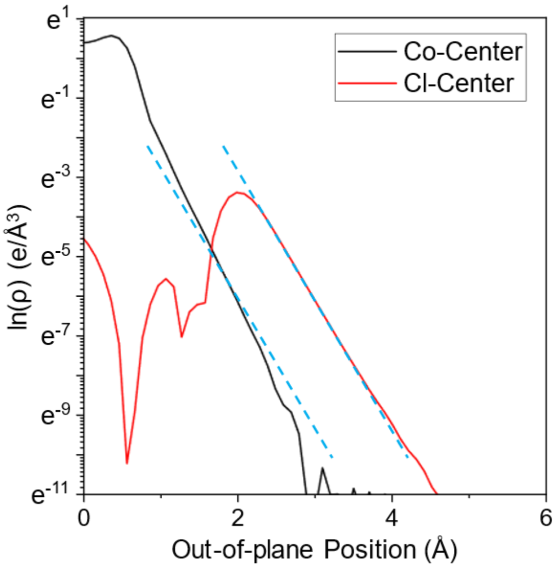

Supplementary Fig. 13. Polaronic charge density of Co-centered (black) and Cl centered (red) states along a line in the out-of-plane direction passing through the polaron center. The position of Co layer is chosen as the zero of horizontal axis. The two blue dashes lines are given as guide to the eye, which shows that the Cl-centered polaronic state has larger charge density and decays slower than Co-centered one in the surface range.

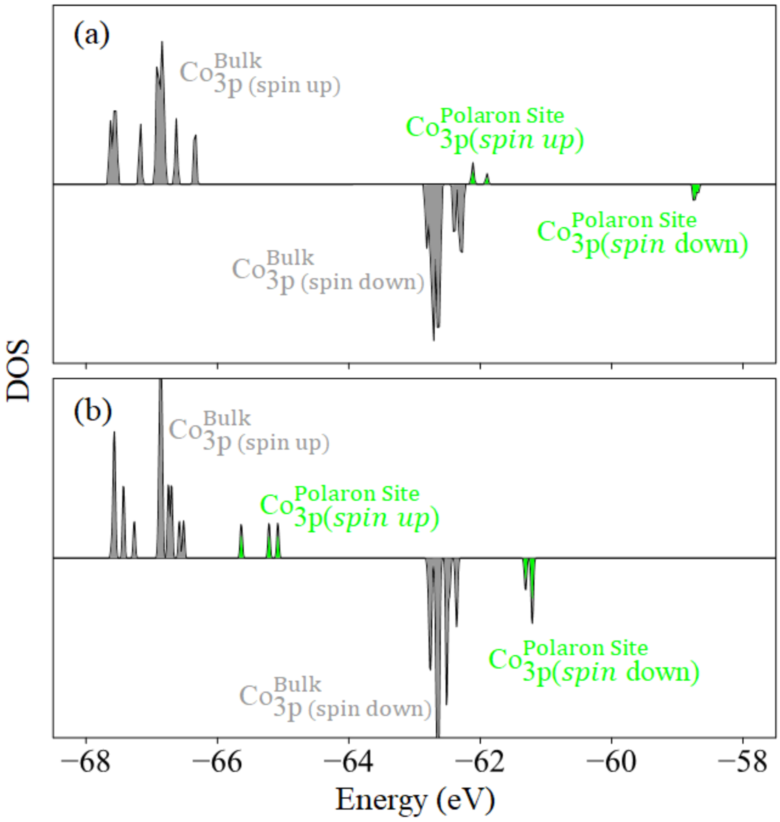

Supplementary Fig. 14. Spin-resolved electronic density of states of (**a**) Co-centered and (**b**) Cl-centered polarons. The green color denotes the Co-3p orbitals contributed by (**a**) the central Co atom of Co-centered polaron and (**b**) the three nearest-neighbor Co atoms in Cl-centered polaron. The Co-3p orbitals of other Co atoms are shown in gray.

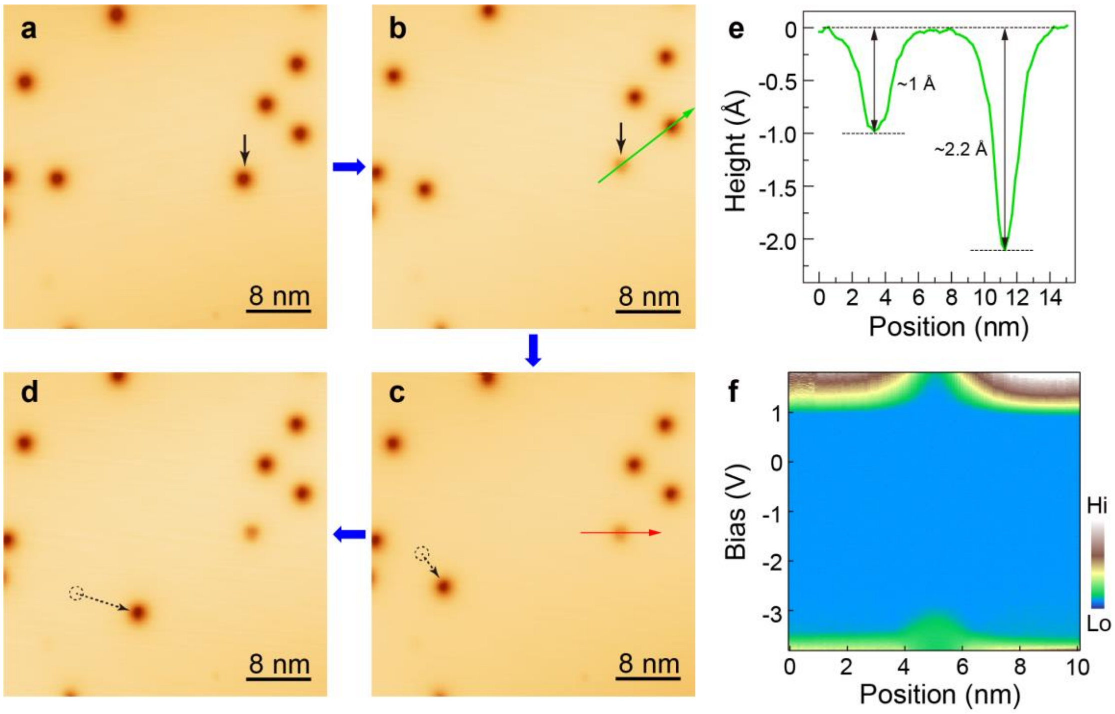

Supplementary Fig. 15 Different types of polarons and their manipulation in monolayer $FeCl_2$/HOPG. (**a-d**) STM images (V_s = 1.5 V, I_t = 10 pA) of the same field of view, showing manipulating sequences of different types of polarons. The STM image in (**a**) are all type-I (up) polarons. When positioning the tip above a type-I (up) polaron (indicated with black arrow) above – 4.2 V, it is converted to a type-I (down) polaron, as shown in (b). (**c, d**) Successive movement of a type-I (up) polaron with the STM tip. Manipulation condition: V_s = 2 V, I_t = 10 pA, moving speed of 100 pm/s. The dashed circles mark the original location of the type-I (up) polaron. (**e**) Line profile of the type-I (down) and type-I (up) polarons taken along the green arrow in (b). (f) 2D conductance spectra measured across a type-I (down) polaron along the red line in (**c**). The upward band bending indicates a localized electron, which is similar to the polarons in $CoCl_2$. Spectroscopic set point: V_s = - 3.8 V, I_t = 50 pA.
